# Insights on the effects of antimicrobial and heavy metal usage on the antimicrobial resistance profiles of pigs based on culture-independent studies

**DOI:** 10.1186/s13567-023-01143-3

**Published:** 2023-02-23

**Authors:** Daniel Ekhlas, Héctor Argüello, Finola C. Leonard, Edgar G. Manzanilla, Catherine M. Burgess

**Affiliations:** 1 Food Safety Department, Teagasc Food Research Centre, Ashtown, Dublin, Ireland; 2 School of Veterinary Medicine, University College Dublin, Dublin, Ireland; 3 Animal Health Department, Veterinary Faculty, Universidad de León, León, Spain; 4 Pig Development Department, Teagasc Moorepark, Fermoy, Co. Cork Ireland

**Keywords:** antimicrobial resistance, metagenomics, qPCR, heavy metals, pigs

## Abstract

Antimicrobial resistance is a global threat to human, animal, and environmental health. In pig production, antimicrobials and heavy metals such as zinc oxide are commonly used for treatment and prevention of disease. Nevertheless, the effects of antimicrobials and heavy metals on the porcine resistome composition and the factors influencing this resistance profile are not fully understood. Advances in technologies to determine the presence of antimicrobial resistance genes in diverse sample types have enabled a more complete understanding of the resistome and the factors which influence its composition. The aim of this review is to provide a greater understanding of the influence of antimicrobial and heavy metal usage on the development and transmission of antimicrobial resistance on pig farms. Furthermore, this review aims to identify additional factors that can affect the porcine resistome. Relevant literature that used high-throughput sequencing or quantitative PCR methods to examine links between antimicrobial resistance and antimicrobial and heavy metal use was identified using a systematic approach with PubMed (NCBI), Scopus (Elsevier), and Web of Science (Clarivate Analytics) databases. In total, 247 unique records were found and 28 publications were identified as eligible for inclusion in this review. Based on these, there is clear evidence that antimicrobial and heavy metal use are positively linked with antimicrobial resistance in pigs. Moreover, associations of genes conferring antimicrobial resistance with mobile genetic elements, the microbiome, and the virome were reported, which were further influenced by the host, the environment, or the treatment itself.

**Supplementary Information:**

The online version contains supplementary material available at 10.1186/s13567-023-01143-3.

## Introduction

Antimicrobial resistance (AMR) is one of the great global concerns of the twenty-first century [1]. The prevalence of AMR in primary production systems such as livestock production is of great concern due to the recognition of the interconnections between human, animal, and environmental health, also termed the “One Health principle” [2]. In pig production, antimicrobials and heavy metals are primarily used for the prevention and treatment of diseases, which are associated with animal morbidity and mortality, reduced performance (i.e., animal growth) which result in high economic losses for the farmer, and health and welfare issues for the animal. However, antimicrobial use (AMU) and heavy metal use (HMU) have been associated with the development and spread of AMR, and global targets for reducing AMU in animal production have been set, as outlined in “The Global Action Plan on Antimicrobial Resistance” and “The OIE Strategy on Antimicrobial Resistance and the Prudent Use of Antimicrobials”, which were launched by the World Health Organization (WHO) and the World Organisation for Animal Health (WOAH) in 2015 and 2016, respectively [3, 4]. Furthermore, the use of heavy metals such as zinc oxide and antimicrobials are also associated with environmental pollution, as they are often not fully absorbed due to their low bioavailability and therefore can be found in high concentrations in livestock manure [5, 6], which is often used as an organic fertilizer on pasture and in crop production, representing a potential risk to environmental and public health.

Based on the requirements of the WHO global action plan, every member state had to develop its own action plan to tackle and prevent the spread and development of AMR. In the latest legislative changes in the EU, the prophylactic use of antimicrobials in livestock production was banned as of January 2022 and the prophylactic use of medicinal levels of zinc oxide was banned from June 2022 based on Regulation (EU) 2019/6 on Veterinary Medicinal Products and Regulation (EU) 2019/4 on Medicated Feed. The aim of these measures is not only to reduce the development and spread of AMR and environmental pollution, but also to suppress the use of antimicrobials in routine farm protocols and additionally promote animal health through farm management, biosecurity, hygiene, immunoprophylaxis and animal welfare measures on farms [7].

Traditional methods to evaluate AMR such as antimicrobial susceptibility tests are dependent on bacterial growth and are mainly restricted to specific culturable species which allows AMR detection only within a limited spectrum. However, antimicrobial and heavy metal resistance are not limited to these bacteria and could be related to non-culturable microorganisms which may play a role in the spread of AMR [8]. Novel approaches in AMR monitoring, such as high-throughput next-generation sequencing and quantitative PCR (qPCR) methods offer further insights and an improved understanding of the prevalence, development, and spread of AMR in association with antimicrobial resistance genes (ARGs). These methods can be used to analyse specific microorganisms, but also to study more complex microbial consortia such as the gut microbiome.

Both methods, qPCR and metagenomics, have their advantages and disadvantages as reviewed by Waseem et al. [9]. New developments in high-throughput qPCR methods such as pre-designed primer sets such as the “Primer set 2.0”, consisting of 384 primer arrays that target ARGs and mobile genetic elements (MGEs), may allow a cost-effective and user-friendly approach in analysing the resistome, compared to metagenomics approaches [10]. Apart from that, qPCR approaches may allow lower detection limits in comparison with metagenomics approaches and allow deeper insights into AMU and HMU associations with AMR [9]. Metagenomics often involves complex analysis pipelines and bioinformatics tools for which skilled personnel are indispensable. While qPCR-based approaches require good knowledge of genes to be targeted as part of the analysis, metagenomics approaches are however more independent and may allow, apart from ARG screening via selected curated databases, the identification of unknown sequences that could also contribute to AMR [9].

The aim of the current review is to summarise the findings of studies investigating the association between AMU and HMU and AMR in pig production using such culture-independent approaches to identify factors and drivers that can impact AMR on pig farms.

## Methodology

### Literature search strategy

A systematic literature search approach was used to identify scientific literature investigating associations between the usage of antimicrobials and heavy metals on commercial pig farms with AMR in pigs. Moreover, this review only included studies that used culture-independent methods such as metagenomics and quantitative PCR, although additional use of culture-dependent methods was not an exclusion criteria.

Literature was identified using the PubMed (NCBI), Scopus (Elsevier), and the Web of Science (Clarivate Analytics) databases. The key questions were formulated according to the PIO (population, intervention, outcomes) criteria. To separate search terms within one PIO group, the Boolean operator “OR” was used (Table 1), thus PIO groups were combined using the Boolean operator “AND”. To further refine the literature search, an additional group was included in the literature search “Additional terms” to exclude all literature that did not present culture-independent based data (Table 1). For the Scopus database, the search was limited to the title, abstract, and keywords to further limit literature results.

**Table 1:** **Search terms used for the PubMed, Scopus, and Web of Science database search engines**

Search term groups	Search terms
Population terms	“Weaning pig*” OR “weanling pig*” OR “weaner pig*” OR “weaning period” OR “weaning stage” OR “weaner stage” OR “weaning phase” OR “piglet*” OR “swine” OR “suckling pig*” OR “farrowing pig*” OR “neonatal pig*” OR “young pig*” OR “nursery pig*” OR “farrowing stage” OR “farrowing period” OR “farrowing phase” OR “finisher pig*” OR “domestic pig*” OR “starter pig*”
Intervention terms	“Zinc*” OR “zinc oxide” OR “ZnO” OR “copper” OR “antimicrobial*” OR “antimicrobial peptide*” OR “antibiotic*” OR “antibacterial*” OR “antibacterial agent*” OR “antimicrobial agent*” OR “prophylactic antimicrobial*” OR “drug therapy” OR “prophylactic antibiotic*” OR “therapeutic antimicrobial*” OR “therapeutic antibiotic*”
Outcome terms	“Antimicrobial resistan*” OR “antibiotic resistan*” OR “drug resistan*” OR “metal resistan*” OR “multiresistan*” OR “multi-drug resistan*” OR “resistome” OR “resistance profile” OR “resistance” OR “microbial resistan*”
Additional terms	“Resistome” OR “metagenom* “ OR “gut resist*”

Wildcards within search terms are indicated by asterisks.

Only records published in English from the 1^st^ of January 2000 until the 10^th^ of February 2022 were included in the results as part of the literature search. All records found were imported into and further processed via EndNote X9 reference management software (Clarivate Analytics; version: X9.3.3). The literature search resulted in the identification of 118, 145, and 180 records, obtained from the Web of Science, Pubmed, and Scopus databases respectively (n = 443 of total records).

### Inclusion and exclusion criteria of papers identified

Prior to literature thinning duplicates were removed using EndNote X9. In total 152 duplicates were removed, leaving 291 records which were further screened via two dependent screening steps to determine their suitability for inclusion in this review. The first screening step was based on available information in the record’s title and abstract, while the second screening step was based on full-text analysis of the records that were not removed in the first screening step.

**Figure 1:**
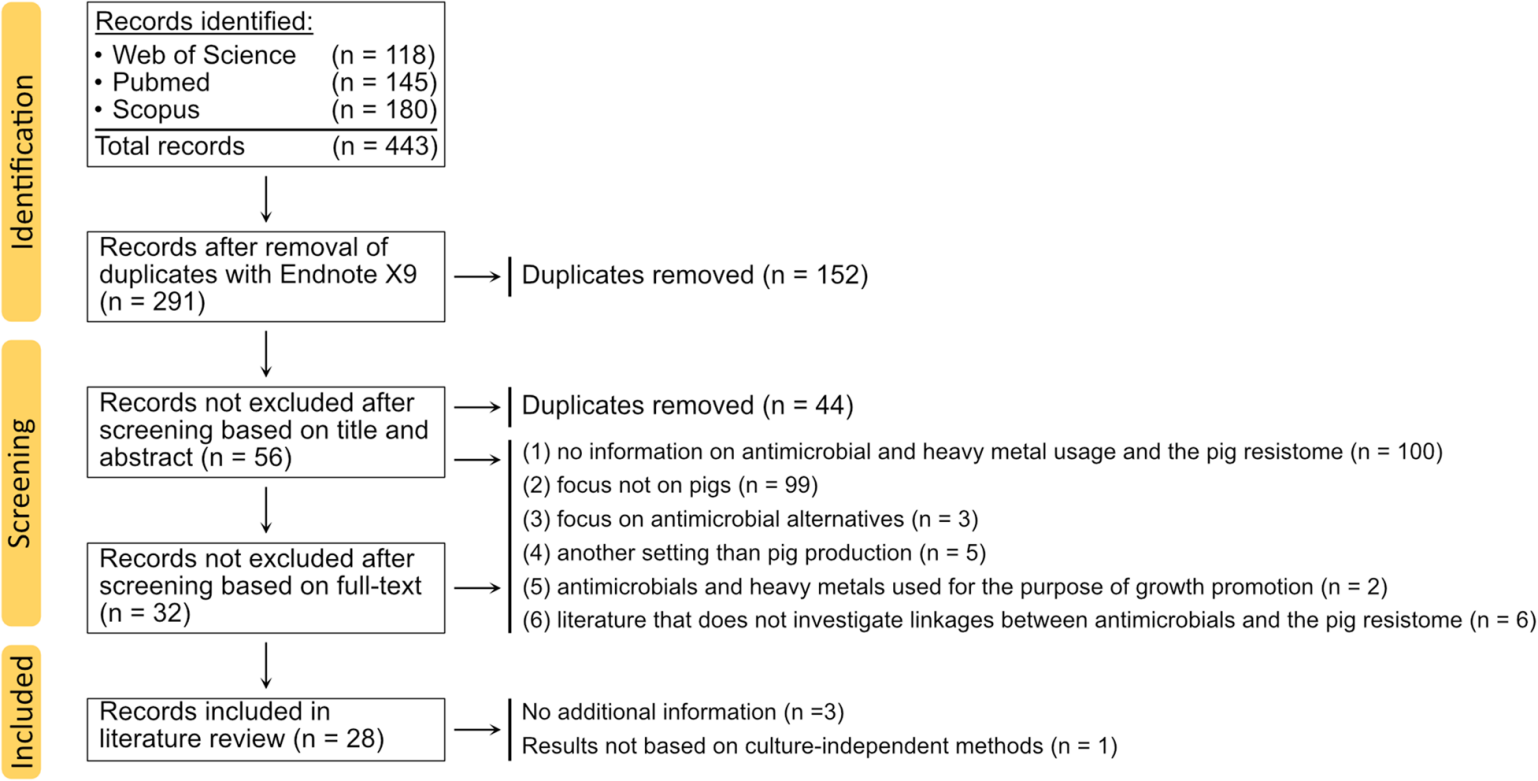
**PRISMA flow-chart representing the literature screening process and selection of eligible literature.**

Duplicates that were not identified by Endnote X9 were removed in the first screening step (*n* = 44). According to the set inclusion criteria, all records that examined how the use of antimicrobials and heavy metals can affect the porcine resistome were classified as suitable. However, literature was excluded in the following cases: (1) no information on antimicrobial and heavy metal usage and the porcine resistome, (2) focus not on pigs, (3) focus on antimicrobial alternatives, (4) a setting other than pig production, (5) antimicrobials and heavy metals used for the purpose of growth promotion, and (6) literature that did not consider associations between antimicrobials and the porcine resistome. After literature screening, 32 records were left, of which 3 reviews were excluded which discussed findings of studies that were already included in the literature selection, and one study that used culture-dependent methods for AMR quantification. Consequently, 28 records remained. Further information on the literature selection can be extracted from the PRISMA flow-chart (Figure 1).

## Results and discussion

### Summary of included literature and their study approaches

**Figure 2:**
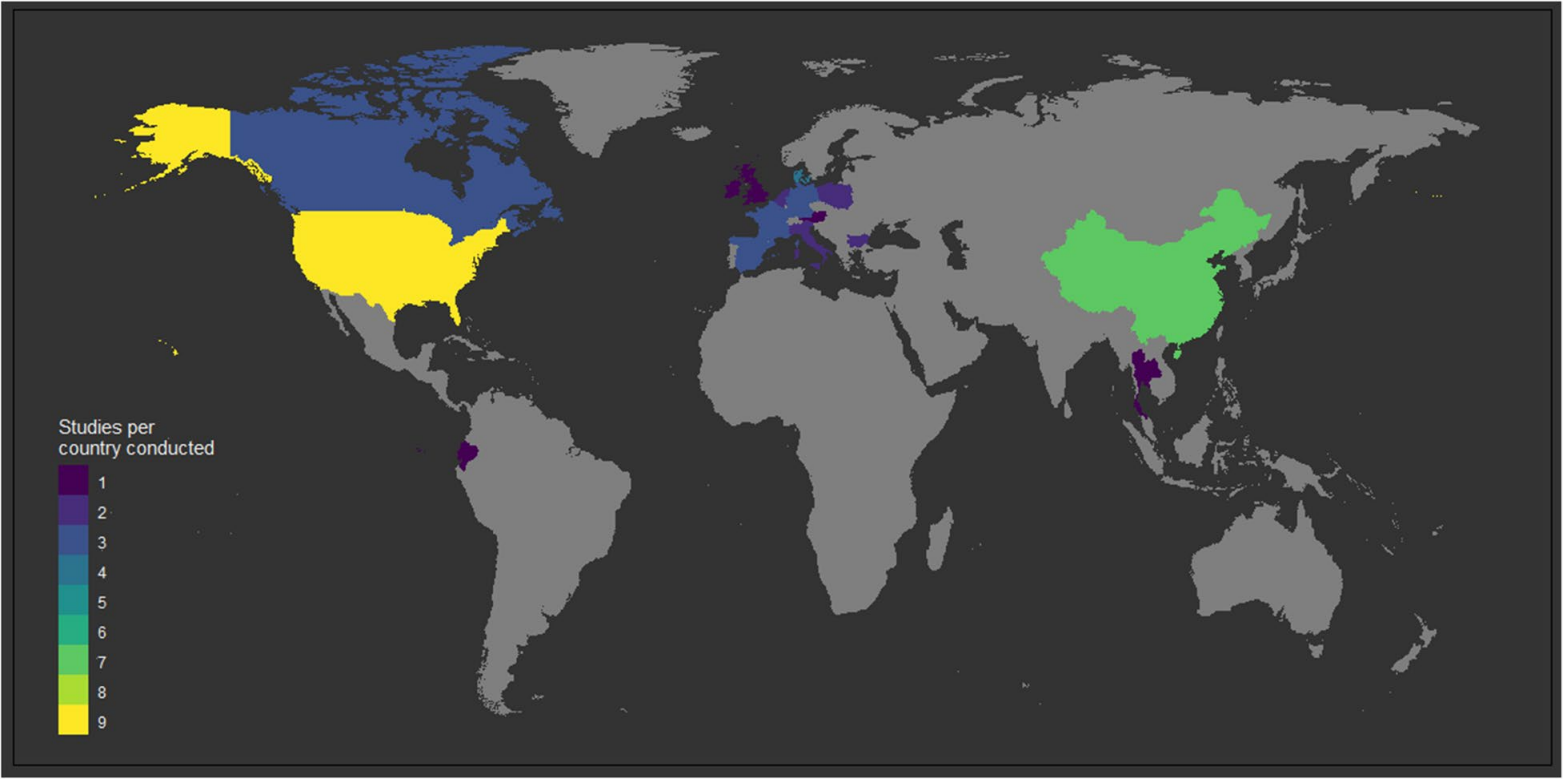
**Geographical distribution of selected studies that were included in this literature review.**

In total, 28 research studies were included in this literature review. Most studies were conducted in a single country (*n* = 24), while four of them were comparisons at an international level. The latter included comparison of a randomized trial in a research facility in the United States of America with AMR on three Chinese pig farms (*n* = 1), comparison of AMR on farms in nine European countries (*n* = 2) and comparison of AMR on a Chinese, a Danish, and a French pig farm (*n* = 1). The majority of single-country studies were conducted in the United States of America (*n* = 8), followed by studies in Europe (n = 6), China (*n* = 5), Canada (*n* = 3), Ecuador (*n* = 1), and Thailand (*n* = 1). No studies based in lower-middle or low income countries were identified, thus a geographical and economical bias as part of this review cannot be excluded. Details regarding the geographical distribution of the identified studies can be seen in Figure 2.

Although the methodologies of the 28 assessed studies differed greatly, the majority of studies (*n* = 19) reported a positive association between AMU or HMU, and AMR (AMU-AMR: n = 18; HMU-AMR: *n* = 5) (Additional file 1). The 9 remaining studies either reported no association between AMU and HMU, and AMR (*n* = 6) or investigated AMR in the absence of AMU and HMU or only resistome development under AMU (n = 3). Studies investigated AMR prevalence by either using a metagenomics (*n* = 17), 16S rRNA gene amplicon sequencing (n = 1) or quantitative PCR (n = 8) approach, or two methods (*n* = 2), as can be additionally extracted from Additional file 1. Of the total 28 studies, fifteen were observational studies and thirteen were randomized control trials.

Interestingly, all sequencing-based studies used short-read sequencing methods such as Illumina (n = 16), 454 pyrosequencing (n = 3), or both (n = 1) for investigating AMR. Potential reasons for this could be the higher costs of long-read sequencing methods, partially related to its reliance on short-read sequencing to compensate for low coverage of long-reads and to overcome high error rates [11, 12]. As a consequence, this is additionally associated with more complex analysis steps [11]. Although these challenges remain, long-read sequencing methods could prevent misassembly of repetitive and highly conserved regions, which would allow deeper and more accurate resolution of metagenomes [11]. Moreover, these methods allow more accurate assignment of functional features such as resistance genes to specific taxa on species level or to MGEs, which is often limited to correlation analysis in short-read sequencing methods. Limitations have been also found in studies that used a qPCR approach. While ten studies used such an approach, only three of them used pre-designed complex primer sets such as the “Primer set 2.0” consisting of several hundred primers for resistance genes and MGEs. The other seven studies used small primer sets which may allow investigation of increases in abundance of specific ARGs, but do not capture the complexity of the metagenomes. Two of these seven studies additionally used short-read sequencing methods to potentially account for this limitation. Associations between AMU and AMR in pig production have been described before in culture-dependent studies [13]. Similar observations have been made for heavy metals. The use of zinc and copper supplementation have been associated with an increase of AMR and its persistence due to cross-resistance (resistance mechanism that confers resistance to multiple compounds) and co-location of ARGs on MGEs conferring resistance to different compounds [13–15]. Furthermore, these mechanisms may facilitate the growth of AMR pathogens during antimicrobial or heavy metal administration due to disruption of the microbial community, as was observed for methicillin-resistant *Staphylococcus aureus* [16, 17].

In this review, the literature identified was divided and summarized into different sections (1) AMR in the absence of AMU and HMU, (2) AMR in association with AMU (see also Additional file 2), and (3) AMR in association with HMU (see also Additional file 3). Moreover, studies were further divided according to the varying methodologies used, namely metagenomics, 16S rRNA gene amplicon sequencing, and qPCR.

### AMR in the absence of AMU and HMU

Two of the 28 selected studies, undertaken in Canada by Holman et al. [18] and in Ireland by Joyce et al. [19] in controlled research facilities, used a shotgun metagenomics approach to investigate the faecal resistome of pigs that received no antimicrobial or heavy metal treatment either for their entire life [18], or since weaning [19] and reported that ARGs conferring resistance to tetracyclines, macrolides, and aminoglycosides were most abundant. Interestingly, resistance to these antimicrobial classes was frequently reported in most of the selected studies, regardless of the treatment provided [20].

Furthermore, both studies reported that the composition of the resistome was strongly influenced by the composition of the microbiota in an age dependent manner, suggesting that core ARGs could be either chromosomally encoded or located on MGEs with a specific host range. This is further discussed later, together with potential drivers of AMR. Holman et al. [18] reported that the abundance of several ARGs conferring resistance to different antimicrobial classes decreased after weaning, except for ARGs conferring tetracycline and MLSB (macrolides, lincosamides and streptogramin B) resistance which were more stable. A possible explanation for these observations could be the host specificity of ARGs, as suggested by their study. Consequently, ARGs with a broader host range, such as those encoding tetracycline and MLSB resistance, are more stable in the porcine resistome despite changes in the microbiome with age or treatment, while ARGs with a narrower host range are more influenced by the composition of the microbiome in an age-dependent manner. This was also seen for two β-lactamase-encoding genes, namely *bla*_CfxA6_ and *bla*_ACI-1_ which were associated with *Prevotella* spp. and increased in abundance together with *Prevotella* spp. post-weaning, as reported by Holman et al. [18, 21]. These findings were also observed by Loayza-Villa et al. [22], who used a qPCR-based approach to investigate the removal of antimicrobial prophylaxis post-weaning (tilmicosin [200 ppm] and colistin [40 ppm] at age 21–28 days; tiamulin [150 ppm] and chlortetracycline [450 ppm] at age 29–70 days; chlortetracycline [450 ppm] at age 70–139 days; additionally trimethoprim-sulfamethoxazole [25 mg/kg/PV] at age 37–40 days and doxycycline [10 mg/kg/PV] at age 45–47 days). In their study no differences in the resistome were observed between antimicrobial treated and untreated pigs post-weaning. Furthermore, similar to Holman et al. [18], they reported a decline of ARGs over time after piglets reached 30 days of age. However, ARGs conferring tetracycline resistance and MGEs were not affected [22].

While the studies of both Loayza-Villa et al. [22] and Holman et al. [18] suggest that shifts in the microbiome post-weaning influence the resistome, it is also plausible that the frequent use of inexpensive antimicrobials in the past, such as tetracycline for growth promotion and for prevention of infections, may have selected for specific resistant bacteria, which persisted in the environment over the years and influence the porcine resistome, as suggested by Wang et al. [23].

As suggested by Joyce et al. [19], the porcine microbiome and its resistome are not only affected by AMU and HMU, but also by external sources of AMR to which pigs are constantly exposed. These external sources can include for instance farm workers, rodents and arthropods, air, dust, soil, surface water, feed and drinking water [24]. External AMR sources may harbour MGEs that are frequently disseminated between bacteria due to genes conferring fitness advantages while co-selecting for ARGs, which allows their acquisition by the porcine resistome [19]. Apart from this, further factors such as genetics, diet, age, and animal health may affect the microbiome, and hence the resistome [25, 26].

### Associations of AMU with AMR prevalence in pigs

#### Metagenomics-based studies

In the first part of this section, AMU associations with AMR are discussed, based on studies investigating the resistome via metagenomics. Probably two of the largest observational studies on pig farm associated AMR in the European Union were conducted by Munk et al. [27] and Van Gompel et al. [28] as part of the EFFORT (Ecology from Farm to Fork Of microbial drug Resistance and Transmission) project, which investigated the porcine resistome by analysing faecal samples of 181 pig farms in 9 different European countries (Belgium, Bulgaria, Denmark, France, Germany, Italy, Netherlands, Poland, and Spain) by using metagenomics. Similar to Joyce et al. [19] and Holman et al. [18], Munk et al. [27] reported that genes encoding tetracycline and macrolide resistance were the most abundant resistance determinants. Furthermore, Munk et al. [27] reported a positive correlation between countries using higher amounts of antimicrobials (e.g. Spain) in relation to other EU countries and AMR prevalence. These observations were also made by Li et al. [29], who investigated the resistome from faecal samples collected on a Chinese farm (high AMU of various antimicrobials), which was further compared with one French (no AMU) and one Danish farm (antimicrobials only used at weaning). Additionally, Li et al. [29] frequently observed the co-occurrence of ARGs with biocide (BRGs) and metal resistance genes (MRGs), potentially carried by members of the *Enterobacteriaceae* family (*Enterobacterales* ord. nov.). Co-occurrence of polymyxin, i.e. colistin resistance, with other ARGs was also observed in their study [29]. However, associations between specific antimicrobial classes used and AMR were not further investigated by Munk et al. [27] but were continued by Van Gompel et al. [28]. In their study, Van Gompel et al. [28], explored the AMU-AMR relationship in different production stages and observed that these associations were only detected in fattening pigs, but not in the early life stages in which AMU is highest. According to the results of Van Gompel et al. [28], macrolide, amphenicol, and tetracycline administration was associated with an increase in abundance of ARGs conferring resistance to these antimicrobials, while this effect was not seen for colistin and aminopenicillin administration. Moreover, their study observed cross-resistance mechanisms that conferred macrolide and lincomycin/spectinomycin resistance and positive correlations between β-lactam administration and amphenicol resistance which suggested potential co-selection mechanisms in response to β-lactam treatment. Furthermore, macrolide resistance was associated with internal biosecurity measures such as the use of disinfectants, which highlights once more the influence of multiple factors on the porcine resistome [28].

Another study by Mencía-Ares et al. [30] reached similar conclusions. In their study, 467 samples derived from pooled faeces, the farm environment, and slurry were collected to compare the porcine resistome of pigs reared in intensive (*n* = 19) and extensive (*n* = 19) pig farms in Spain by using a metagenomics approach. Extensive pig farms, i.e. traditional outdoor farming of Iberian pigs, were described in the study as more sustainable and eco-friendly, with constrained use of antimicrobials and a lower animal density compared to intensive farms [30]. Based on their results, they observed correlations between tetracycline, aminoglycoside, MLSP (macrolide, lincosamide, streptogramin, pleuromutilin) use with ARGs conferring resistance to these agents. Moreover, their study reported that ARG richness was higher on intensive than extensive farms, which also applied to the diversity of ARGs conferring tetracycline resistance, which were mostly associated with *Bacteroidaceae* (*tetQ*), or members of the phyla *Bacillota* corrig. phyl. nov. (formerly: *Firmicutes*), including the family *Streptococcaceae* (*tetL* and *tetM*). In contrast, the diversity of ARGs conferring resistance to β-lactams was higher in extensive farms than intensive farms [30]. Resistance genes to MLSP and oxazolidinone were mostly associated with *Bacillaceae*, *Streptococcaceae* (*optrA*), and *Peptostreptococcaceae* (*cfr(c)*) families. Furthermore, their study reported that 120 bacterial families were associated with 94% of total assigned ARGs. Co-selection and cross-resistance were additionally observed. For instance, the administration of phenicols, tetracyclines, or MLP (macrolide, lincosamide, streptogramin) was positively associated with resistance to oxazolidinone and other antimicrobial classes. However, no positive correlation between β-lactam administration and β-lactam resistance was observed [30]. Although the authors did not provide an explanation for this observation, they observed a lower abundance of β-lactam ARGs on MGEs compared to ARGs of other antimicrobial classes. Thus, further research is necessary to explain the effects of AMU from different classes on AMR spread via MGEs and its contribution to the total observed AMR in metagenomic studies.

Associations between specific ARGs and specific taxa were also identified by a metagenomics-based study of Suriyaphol et al. [31], who investigated the effect of weaning on the porcine resistome and microbiome. Their study reported that the highest observed abundance of *tet(40)*, *tet(W)*, and *mefA*, conferring tetracycline and macrolide resistance respectively, was in association with *Prevotella* spp. abundance at 8 days post-weaning. Furthermore, at 3 days post-weaning aminoglycoside resistance was predominantly present in these piglets, which was possibly associated with *Escherichia coli*. However, it is important to mention that in the study of Suriyaphol et al. [31] piglets and sows were prophylactically treated with different antimicrobials either in-feed or intramuscularly. Nevertheless, no linkages between AMU and AMR were drawn in their study.

Similar to the study of Mencía-Ares et al. [30], two studies by Chekabab et al. [32] reported that removal of AMU resulted in decreased prevalence of AMR. For example, they observed that pigs raised without AMU had an overall reduction in genes encoding resistance to aminoglycosides, macrolides, phenicols, and tetracyclines in faecal samples. Comparably, Chekabab et al. [33] observed decreased abundance of ARGs conferring multidrug resistance, which seemed to be associated with reduced use of antifolates and β-lactams. However, increased resistance to aminoglycosides was observed in the AMU-free group. Interestingly, differences in abundance of pathogens were reported in both studies; while the first study observed a higher prevalence of pathogenic *Bacillota* corrig. phyl. nov. (formerly: *Firmicutes*) in the AMU-free group, the latter study reported a decrease in overall pathogen prevalence on non-AMU farms [32, 33].

Interestingly, one of the most tested antimicrobials within the randomized metagenomics studies (*n* = 8) was tetracycline in various forms, such as oxytetracycline (*n* = 1) or ASP250 (mix of chlortetracycline [100 mg/kg], sulfamethazine [100 mg/kg], and penicillin [50 mg/kg]): *n* = 3; Oxytetracycline mix: *n* = 1). Considering that tetracycline is one of the most commonly used antimicrobials in pig production on a global scale, it is not surprising that special attention has been given to the effect of tetracycline on the porcine resistome [34]. Metagenomic studies that either investigated the effects of tetracycline alone or in combination with other antimicrobials frequently observed changes in the resistome. For instance, another study by Munk et al. [35] compared farms with low and high AMU in Denmark and observed that tetracycline use resulted in increased abundance of *tet(44)*. Furthermore, they reported that macrolide use was associated with an increase of *erm(B)* and *erm(G)* abundance. Aminoglycoside use increased abundance of the aminoglycoside resistance genes *apmA, ant(6)*’-I and *str(B)*, as well as the tetracycline resistance gene *tet(44)*, which suggested potential co-selection of *tet(44)* under aminoglycoside treatment. Comparably, Ghanbari et al. [36] observed an increase of overall ARG abundance in response to 7 days of oxytetracycline treatment, which mainly included genes conferring resistance to tetracycline, β-lactams, and multidrug resistance. Moreover, the effects of oxytetracycline on the resistome were still visible 14 days after antimicrobial withdrawal. Similar findings were made by Mu et al. [37], who investigated the effect of antimicrobial treatment in the early life of piglets, using a 16S rRNA gene amplicon sequencing approach together with PICRUSt analysis in their study. Piglets were fed a mix of oxytetracycline calcium, kitasamycin (macrolide antibiotic), and olaquindox (quindoxin antibiotic) from 7 days of age. Treatment was strongly associated with an increased abundance of the *emrK* and *emrY* multidrug resistance genes which confer increased tetracycline resistance [37]. In two independent studies examining the effects of ASP250 on the pig resistome, an increase of ARGs conferring resistance to β-lactams, sulphonamides, but also to aminoglycosides was observed [38, 39]. Furthermore, Looft et al. [38] reported dramatic shifts of the resistome in response to 3-weeks of ASP250 treatment, with an increase in ARG diversity and abundance. An association between tetracycline resistance and ASP250 treatment which contains chlortetracycline, was not observed in either study.

An association between the use of sulphonamides with sulphonamide resistance has been described by Wang et al. [23]. In their study, the faecal resistome of pigs in four industrialized pig units in China was compared. Although no detailed report of AMU on farms was provided, higher resistance to sulfamethoxazole-trimethoprim was observed in the faecal resistome of pigs in three intensive units that used sulphonamides widely, while the pigs in the other unit which was the only unit using ciprofloxacin showed an increased abundance of ARGs conferring ciprofloxacin resistance (*qnrB*, *qnrS1*, *qnrS2*) in their faeces. However, differences between pig units could not be solely explained by AMU, suggesting that AMR in pig units is not only associated with AMU but also influenced by factors such as internal biosecurity protocols, AMU records and persistence of AMR bacteria in the environment [23]. Other relevant factors could include (1) the co-location of ARGs and MRGs on MGEs, allowing the co-selection of multiple resistance genes under AMU, (2) cross-resistance mechanisms, and (3) co-regulatory mechanisms, which would further allow the co-selection for AMR patterns [15]. Interestingly, Wang et al. [23] observed a high abundance of multidrug resistance genes in association with AMU at the four pig units, which may allow co-selection. Their study however did not investigate MGEs or the co-occurrence of ARGs and MRGs, which could have potentially explained observations that were made in the study.

Separately, a metagenomics-based study by Zeineldin et al. [40] investigated the effect of perinatal administered tulathromycin, i.e. a bacteriostatic macrolide, by a single dose antimicrobial injection in new born pigs on the porcine resistome. Interestingly, no effect on the porcine resistome due to tulathromycin was observed up to 20 days post-treatment in piglets.

#### Quantitative-PCR-based studies

Associations between AMU and AMR have been also reported in studies using qPCR. For instance, Yue et al. [41] quantified antimicrobial and heavy metal concentrations in 107 faecal samples of pigs collected from 62 farms by using LC–MS (liquid chromatography-triple quadrupole mass spectrometry), ICP-MS (inductively coupled plasma mass spectrometry), and atomic fluorescence spectrometry, and described further correlations of antimicrobial and heavy metal pollution with AMR. According to their results, the use of tetracyclines, fluoroquinolones, and macrolides did not affect the porcine resistome. However, sulphonamides positively correlated with resistance genes to sulphonamides but also macrolides, i.e. with the *ermX* resistance gene, suggesting the co-location of *ermX* on MGEs with ARGs conferring resistance to sulphonamide. The phyla *Pseudomonadota* corrig. phyl. nov. (formerly: *Proteobacteria*), *Bacillota* corrig. phyl. nov. (formerly: *Firmicutes*), and *Actinomycetota* corrig. phyl. nov. (formerly: *Actinobacteria*) were identified as potential carriers of these ARGs and MGEs, which is in accordance with previous studies such as those of Mencía-Ares et al. [30, 41]. Similarly, changes in multiple ARGs as a result of treatment due to co-location of these ARGs on MGEs was described by Muurinen et al. [42]. In their study, in which piglets were treated for 33 days with either carbadox, zinc oxide, or copper sulphate (at therapeutic levels), or mushroom powder and compared to a non-treated piglet group the authors reported that the abundance of ARGs and MGEs in control and treatment groups were nearly the same. However, some differences between treatments were observed. Animals treated with carbadox showed increased abundance of *vat(E)* (confers resistance to streptogramin A)*,* but decreased abundance of *tetM*, *tetW*, *tet(32)*, *erm(B)*, and *ermT.* Likewise, a study by Pollock et al. [43] which used a metagenomics approach with three sampling points and quantified five targeted ARGs via qPCR (*tetB*, *tetQ*, *ermA*, *ermB*, and *dfrA1*) and in which different antimicrobials, acidified water, and zinc were administered throughout the production cycle of the pigs, did not find any positive association between chlortetracycline treatment and abundance of the studied ARGs conferring resistance to tetracycline. Instead, they observed decreases of *tetB* but no changes in the abundance of *tetQ*, in association with chlortetracycline use. Additionally, tylosin administration in their study did not affect these 5 ARGs within the full production cycle. The authors suggested that the failure to detect effects of AMU on AMR may have been due to an already saturated ARG pool as a result of previous high AMU on the studied farm, which highlights again the importance of external AMR sources on farms.

Changes in *ermB* and *tetW* have been further described by Zeineldin et al., who investigated the effects of a single dose antimicrobial injection in new born pigs and observed an increase in ARG richness with age that was independent of treatment [44]. In contrast to their previous study [40], an increase of abundance of *ermB* and *tetW* in response to single dose tulathromycin and procaine penicillin G perinatal metaphylaxis was observed, suggesting that these ARGs may be co-located on MGEs [44]. Single dose treatment with the crystalline free acid of ceftiofur and ceftiofur hydrochloride did not affect the levels of the seven ARGs (*ermB*, *tetW*, *tetO*, *tetC*, *sul1*, *sul2,* and *bla*_CTX-M_) which were analysed in their study. Interestingly, Zeineldin et al. [44] further demonstrated the presence of these ARGs in neonatal piglets. Furthermore, their study observed an increase in mortality of tulathromycin treated piglets between 15 to 20 days of age and increased abundance of pathogens in antimicrobial treated piglets. In summary, studies to date suggest that the effects of perinatal antimicrobial prophylaxis on the porcine microbiome and resistome do not follow a clear pattern and can potentially have counterproductive effects as was demonstrated by these two studies [40, 44].

Changes in ARGs conferring tetracycline resistance have been also described by Agga et al. [45]. In their study, piglets of 5 weeks of age were continuously treated with in-feed chlortetracycline, copper, or a combination of both. According to their study, increases of *tetA* abundance in pigs treated for three weeks with chlortetracycline in combination with or without copper at therapeutic concentrations were observed. Furthermore, *tetA* positively correlated with *bla*_CMY-2_ implicating a co-selection for ARGs conferring β-lactam resistance with ARGs conferring tetracycline resistance on exposure to chlortetracycline [45]. Other tetracycline-combined treatments such as ASP250 were also reported to be associated with an increase in ARGs conferring β-lactam resistance. For instance, a study by Johnson, et al. [46] which used a cluster analysis and a qPCR-based approach for describing the resistome of pigs in a research facility in the U.S. in comparison with Chinese farms, observed increases in *bla*_TEM_, *sul1,* and *aph(3’’)-Ib* in response to ASP250 treatment, which was administered in the American research facility. Similar to previous mentioned studies, they concluded the co-location of these three genes may have resulted in their increase in abundance in response to ASP250 with *E. coli* as their carrier, which would explain the increase of *aph(3’’)-Ib* in the absence of selective pressure [46].

A study conducted in 5 pig units in China by Xia et al. [47] which investigated the prevalence of the *mcr-1* colistin resistance gene by taking environmental and manure samples before and after the ban on colistin in China, observed a positive correlation between colistin use and *mcr-1* abundance. Thus, *mcr-1* prevalence decreased after the colistin ban. In addition, *mcr-1* was strongly correlated with the genus *Escherichia* on two farms in that study. Persistence of *mcr-1* on some farms after the colistin ban may be due to co-selection with other ARGs or environmental influences, which could explain why no correlations of colistin administration with colistin resistance genes were observed by Van Gompel et al. [28]. Furthermore, considering that colistin resistance is much more prevalent in Asia than Europe, as reported by a review of Dadashi, et al. [48], associations of colistin resistance with colistin use may be less visible in European studies.

### Associations of HMU with AMR prevalence in pigs

#### Metagenomics-based studies

In contrast to associations between AMU and AMR, much less has been published to date about HMU and AMR. Only two studies were found in this review that used a metagenomics approach to investigate effects of heavy metals on the porcine resistome.

One of these studies was conducted by Pieper et al. [49], who investigated the effects of the use of dietary and therapeutic concentrations of zinc in different chemical forms on the porcine resistome. According to their findings, therapeutic concentrations of zinc oxide were associated with an increase in *aph(3’’)-Ib*, which confers resistance to aminoglycosides. Additionally, zinc treatment was associated with an increase of *bla*_ROB_, *pat(A)*, *lnu(C)*, and *arnA*, conferring resistance to β-lactams, phosphinothricin, MLSP, and polymyxin respectively*.* Furthermore, dietary concentrations of zinc oxide and zinc lysate were associated with an increase in *cfxA2* and *erm(G).* These findings suggested co-selection of ARGs with zinc treatment.

The second study by Pollock et al. [43] reported that acidified water in combination with zinc (2500 ppm in feed; not specified in which form) administered to young piglets increased the abundance of ARGs conferring cross-resistance. This may represent another mechanism, which could explain observed changes in the resistome in association with zinc usage. Nevertheless, considering their power more metagenomics-based studies are necessary to further investigate HMU and AMR associations on pig farms.

#### Quantitative-PCR-based studies

Associations of zinc oxide and copper sulphate with AMR were reported by Muurinen, et al. [42] as part of their qPCR-based study. Although the resistome compositions of non-treated piglets and piglets treated for 33 days with either zinc oxide or copper sulphate (at therapeutic levels) were similar, animals receiving zinc oxide or copper sulphate showed increases of *tetM* and *vat(E).* Comparably, associations between tetracycline resistance genes and copper sulphate treatment were reported by Agga et al. [45], who reported that high dietary concentrations of copper sulphate [125 mg/kg of feed] were associated with increases of *tetP* and *tetB* but with a decrease of *tetA* and *pcoD* (confers copper resistance) abundance, although it was unclear why copper sulphate treatment affected tetracycline resistance genes differently and resulted in a decrease of *pcoD*.

A study by Zhao et al. [50] quantified heavy metal concentrations in administered feed by using ICP-MS, investigating their effect on the porcine resistome through the analysis of faecal samples derived from two low-AMU farms and one high-AMU pig farm in China. Their study observed a correlation of copper and arsenic usage with ARG diversity, suggesting their role as co-selective agents. A potential explanation for these observations could be the co-location of ARGs with MRGs or on MGEs, such as seen for the *Salmonella* Genomic Island 4 (SGI-4). This integrative conjugative element has been described before in pig production and has been frequently reported in *Salmonella enterica* serovar 4,[5],12:i:-, often carrying multidrug resistance modules such as R-type ASSuT, encoding resistance for ampicillin, streptomycin, sulphonamides, and tetracycline [51, 52]. Moreover, serovar 4,[5],12:i:- is one of the most common causes of salmonellosis worldwide, with pigs being one of the suggested main vectors [53–55]. This again highlights the risk posed by the interconnections of ARGs with MRGs for human health and potential drivers of AMR dissemination in pig production.

### Known drivers and mechanisms of AMR determinants in pigs

Although the majority of studies using a metagenomics- or qPCR-approach reported associations between AMU and HMU and AMR, the observations were highly variable. One explanation for heterogeneity of changes in the resistome in response to heavy metal or antimicrobial treatment is a possible influence of the microbiome composition on the resistome [37]. As mentioned before, Mencía-Ares et al. [30] associated 94% of total assigned ARG reads with 120 different families. Furthermore, correlations between ARGs and specific taxa have been reported by several studies included in this review [29, 30, 41]. Zhao et al. [50] reported that the microbiome, as well as AMU and HMU, explained 71.6% of variance of observed ARG patterns, suggesting a possible association between the microbiome and the resistome. Johnson et al. [46], performed co-occurrence network analysis of ARGs, MGEs, and taxonomic analysis at genus level and observed similar. An important factor influencing microbiome and resistome relationship is the age of the pigs during treatment and resistome assessment. As discussed by Agga et al. [45], the age of pigs correlated with the observed microbiome composition and the abundance of *tetA* and *tetB*. This may also explain why some studies such as that of Pollock et al. [43] did not report any effect of AMU on the microbiome, or associations between microbiome and resistome.

Interestingly, Muurinen et al. [42] observed associations between the microbiome and resistome only in the control group, but not in the treatment groups. Based on their results, they suggested that co-occurrence of ARGs on MGEs in combination with increased AMR mobility due to treatment could decouple associations between the microbiome and the resistome. Similarly, Yue et al. [41] observed an increased spread of ARGs via horizontal gene transfer (HGT), predominantly mediated by *intl1* and *ISCR1,* and vertical gene transfer (VGT) in response to sulphonamide treatment. In contrast, Zhao et al. [50] reported that HGT had little effect on the resistome. Mencía-Ares, et al. [30] observed increased abundance of MGEs on intensive farms, but no differences in HGT events compared to extensive farms, which is in contrast to the microbiome-resistome decoupling hypothesis suggested by Muurinen, et al. [42]. Taking all this together, it is likely that the resistome is shaped to a greater or lesser extent by the microbiome and HGT and VGT in response to antimicrobial and heavy metal treatment, as observed by Munk et al. [27] and Pieper et al. [49]. Nevertheless, it is also important to consider which ARGs are present and co-located on transferred MGEs. Thus, co-selection may play an important role in terms of microbiome and resistome stability in the presence and absence of heavy metals and antimicrobials. This was suggested by Johnson et al. [46], who observed higher stability of some ARGs during composting. More stable antimicrobials, such as sulphonamides, or heavy metals may persist during the composting process and thereby select for ARGs conferring resistance to them. Consequently, co-location of ARGs on MGEs may allow persistence of ARGs to further antimicrobials and heavy metals [46]. Furthermore, co-location of ARGs may also be the reason why some authors such as Muurinen et al. [42] did not observe major differences in antimicrobial, heavy metal, and alternative treatments. This highlights the problem of AMR persistence on pig farms and may indicate that the withdrawal of antimicrobials and heavy metals from common farm practices may not lead to reduced AMR, since other practices such as the use of disinfectants may support the further persistence of AMR [28].

The virome constitutes another factor that is seldom described in the literature as of yet, but potentially affects the resistome, or more precisely the bacteriophageome. For instance, lysogenic bacteriophages usually infect bacteria, integrate their genetic material into the bacteria chromosome and become dormant until the host bacterium experiences stress. In response to host stress, the phage switches to the lytic cycle and forces the host to reproduce and assemble new bacteriophages, which includes the excision of the prophage from the host chromosome for packaging. Afterwards, the host is killed by lysis [56]. During the excision of the prophage, bacterial DNA such as ARGs can be included into the phage capsid by accident and transferred via transduction to other bacteria, thus facilitating the spread of AMR [56]. Two studies included in this review investigated the importance of transduction on the spread of AMR, the bacteriophage associated resistome, and the effects of carbadox and ASP250 treatment on both of them. Both studies observed that most bacteriophages identified as part of the virome were associated with the phyla *Bacillota* corrig. phyl. nov. (formerly: *Firmicutes*) [57, 58]. Although both studies reported single but also multiple ARGs on viral contigs, increases in ARG spread in response to treatment were not observed. Johnson et al. [58] reported an overlap of microbial plasmids and the phage resistome which suggests the potential for AMR spread via transduction. Furthermore, Allen et al. [57] observed a higher abundance of bacteriophages in response to ASP250 treatment although not to carbadox. Their study suggested that the relationship between bacteriophages and the microbiome could be described via the kill-the-winner hypothesis, which allows the phage-host diversity and abundance to be maintained under antimicrobial treatment. Current knowledge on bacteriophages and the virome and their role in AMR is very much still in development, but presented evidence suggests that the virome could be an important link in our understanding of the resistome.

## Future perspectives in culture-independent methods to study AMR in livestock production

The investigation of AMR in livestock production via high-throughput methods, such as qPCR and metagenomics approaches has increased within the last decade. Reasons for this are the decreasing costs and further developments of next-generation sequencing and qPCR methods in recent years, which has allowed increased sample sizes, but also greater analysis depth of various samples deriving from different environments, humans, and animals [9, 59]. The development of tools for DNA extraction, sequencing, bioinformatics analysis of metagenomics data, and curated databases have also rapidly evolved which has contributed further to the increased usage of such high-throughput methods [60].

However, despite these massive developments over a short period of time, these high-throughput methods still face many limitations, such as the loss of species or contamination of samples during DNA extraction [61]. Additionally, the reported presence of an ARG via qPCR or metagenomics does not mean that the gene is necessarily expressed [30, 62]. Another limitation that was observed when comparing all studies in this review was that there was no gold standard in either reporting AMU and HMU, animal age, treatment, sample size, or in sample analysis (including metagenomics pipelines and databases). Thus, although the majority of studies reported AMU and HMU associations with AMR, it is unclear if differing experimental set-ups (including observational vs. randomized studies) may have affected observations that were made. As discussed in a review by Hirst et al. [63], randomization of animal trials can reduce detection bias and the exaggeration of research findings. Randomized trials permit association of the intervention with causality, but thereby may exclude other factors such as the environment, in-house farm protocol procedures, and rare adverse effects [64]. Apart from that, randomized trials may face ethical issues such as the selection of treatment agents and treatment time, but also the selection of placebo or control groups, considering that the placebo or control group is potentially denied optimal treatment [65]. On the contrary, observational animal trials may account for additional factors in farm environments. However, causality cannot be deduced from such studies; only associations between treatment and results can be explored, with a consequent risk of bias [64].

Another still unsolved challenge is the accurate ascription of ARGs and MRGs to specific taxa. A number of studies (*n* = 7) tried to address this by calculating correlations. Mencía-Ares, et al. [30] on the other hand used a different approach, in which they taxonomically assigned contigs and simultaneously analysed the same contigs for ARGs. Although the correlation analysis approach can help in determining potential carrier taxa of ARGs, its resolution declines with decreasing the taxonomic level to analyse and can easily become computationally intensive [66]. Therefore, these results have to be interpreted carefully. However, one additional advantage of this approach is that qPCR and 16S rRNA gene amplicon sequencing can be easily combined, as conducted by Xia et al. [47], and may allow a more accurate resolution of correlations between taxa, MGEs, and ARGs due to potential detection of low abundant genetic elements [9, 67]. Such elements may be omitted when using a metagenomics approach. Nevertheless, both approaches are limited to the knowledge currently available. Thus, while qPCR approaches are limited to specific targets, metagenomics results are limited, and may vary, based on the databases used. Newer approaches in metagenomics such as assignment of extrachromosomal contigs [68], identification of HGT events [69], and the construction of metagenomics-assembled genomes [70] seem promising in allowing a deeper and more accurate analysis of AMR spread and development. However these methods are still in their early stages.

It is necessary to standardize future resistome studies to allow generation of accurate, precise, and comparable results. Moreover, studies should further investigate potential associations between AMR with the microbiome, virome, and MGEs to provide deeper insights into the development and spread of AMR in the primary production sector.

## Conclusions

**Figure 3:**
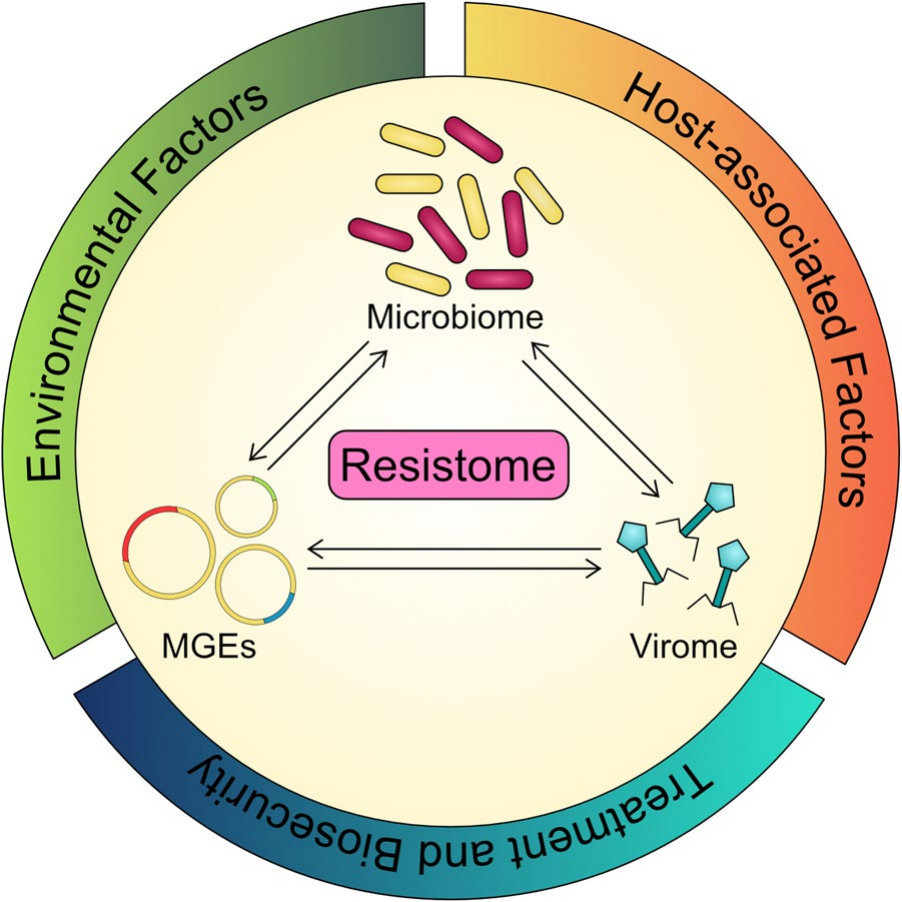
**Interconnections between different factors affecting the porcine resistome.**

Antimicrobial resistance in animal production is of great concern worldwide due to possible impacts on animal, human, and also environmental health. On pig farms, antimicrobials and heavy metals are generally used for treatment or the prevention of disease. However, national and international action plans against AMR spread and development, such as seen in Europe, ban and / or limit the use of antimicrobials and heavy metals on pig farms based on possible associations between AMU and HMU, and AMR. In this systematic literature review, clear evidence was found that AMR is positively associated with AMU and HMU. Moreover, this literature review highlights the usefulness of culture-independent methods in AMR studies. Although antimicrobial or heavy metal treatment do not always result in AMR development, increases in AMR associated with treatment were observed in most of the 28 studies. Reasons for observed heterogeneity in results include the complexity of AMR, which in some instances may be more associated with the microbiome, the virome, or with MGEs (Figure 3).

Interactions between these three groups of factors that influence AMR can result in different observations in response to AMU and HMU. Moreover, these factors are further affected by aspects such as host genetics and age, the environmental microbiome (which in turn is affected by cleaning and biosecurity), and feed, water, as well as the kind of antimicrobial compound or heavy metal used. Due to this complexity, comparison of farms or pig production facilities with each other to find a common solution against AMR, is extremely challenging. Consequently, each farm or pig production facility will need its own suite of measures to address AMR spread and development by reducing AMU and HMU, while maintaining animal health and welfare. Future studies should include analysis of the microbiome of different elements of the production cycle, the virome of the host, and MGEs in their analysis and try to set observations into context with environmental and host-derived factors to obtain deeper insights on the interplay between AMR key factors. Furthermore, a standardized protocol for analysis via e.g. metagenomics could be helpful in the future to allow better comparability of studies.

## Supplementary Information

**Additional file 1. Methodologies used by studies investigating AMU and HMU associations with AMR in pig production.** Table summarizing methodologies used by studies.

**Additional file 2. Key observations indicating associations between AMU and AMR in identified studies undertaken in pig production.** Table of found AMU associations with AMR in pig production.

**Additional file 3. Key observations indicating associations between HMU and AMR in identified studies undertaken in pig production.** Table of found HMU associations with AMR in pig production.

## Data Availability

The datasets supporting the conclusions of this article are included within the article and its additional files.
